# Supplementary material for: Private practitioners’ practices for tuberculosis management in a city largely served by the private health sector in Uganda
Source: PLoS One. 2024 Jan 23;19(1):e0296422. doi: 10.1371/journal.pone.0296422 (PMC10805318; doi:10.1371/journal.pone.0296422)
Supplement: Attachment — Submitted filename: Response to Reviewers.doc [file pone.0296422.s002.doc]

**REBUTTAL LETTER RESPONDING TO COMMENTS RAISED BY PLOS ONE EDITORIAL TEAM.**

23^rd^ October, 2023

To

Lara Vojnov

Academic Editor

PLOS ONE

Dear Editor,

**Reference: PONE-D-22-31914R1**

We thank you for reviewing and inviting us to submit a revised version of our research manuscript titled, ‘Private Practitioners’ practices for tuberculosis management in a city largely served by the private health sector in Uganda’.

We have worked on the comments and made all the effort to ensure that the format of the text and references are standard.

Kindly receive our rebuttal letter responding to each of the comments raised by the reviewers. We have submitted the revised manuscript with track changes as well as a clean copy.

**Responses to comments from Reviewer #1**

Thank you for encouraging us to improve our manuscript and for taking time to review and make it better.

**Comment #1**: Line 52 - not registered - but 1.6m are estimated

Response: We changed the wording from registered to estimated.
**Comment #2:** 62 - spacing issue

Response: We have formatted the spacing.
**Comment #3**: 88 - strange to say approx and then sch a specific number 1442... maybe better to say estimated? or registered? also a spacing issue

Response: We have formatted the wording and the spacing.
**Comment #4**: 89 - can you define what TB/HIV services are? treatment provision? diagnosis? referral?

Response: TB/HIV services here refer to provision of both diagnostic and treatment services for TB and HIV patients.
**Comment #5**: Lines 114, 121 and 122 - I had a hard time understanding why you say estimate of 24% from the literature and then use much higher estimates of 69 and 57% with other refs - but that cannot all be true since 24% overall is lower than both the others.

Response: We used the 24% from a study done in Uganda many years back to calculate the sample size using Kish and Leslie formula. The study used the sample size of 630 which we got using this proportion of 24% and catering for non-response. The other proportions of 68% and 57% from a different study were used to calculate the sample size formula for comparing two proportions. But we never considered this sample size since it was smaller. We have removed this from the text since it creates some confusion.
**Comment #6**: 157-8 - please cite the literature you mention.

Response: We inserted the citation.
**Comment #7**: 190 is the committee really just School Of Medicine? Not Kampala or something else?

Response: It is Makerere University School of Medicine Research Ethics committee. We have corrected this in the text.
**Comment #8**: 198 IQR is not correct/incomplete)

Response: We have corrected this one.
**Comment #9:** 201 and 203 - no need to have separate paragraphs here with one sentence each.
203 - very specific to say approximately with 16.8% - Overall?

Response: We have combined the sentences into one paragraph and replaced ‘approximately’ with ‘overall’.
**Comment #10:** 379 - representative for Kampala.

Response: Added Kampala to the sentence.
**Comment #11**: Table 1 - I think it would be good to describe a little more how many PP actually saw a person with presumptive TB - and document that - as this is important when you are making a case this is a population that can add a lot of TB detection and diagnosis to the NTP. It seems that at least 25% didn’t see any which leads me to my only bigger point for the discussion.

Response: Thank you for this important insight into the data, indeed it raises a major point of discussion. We have added a narrative to table 1 to include this information.

**Comment #12**: The discussion is still much too long and should be reduced substantially - it is more than 5 pages now and this should be reduced to 3 pages highlighting the important parts of the findings and contrasting them with other work. It is not right to link poor practices in Kampala to not being able to meet global TB targets. The link is not that strong, and it seems a stretch to make such a connection. TB efforts can be strengthened and more poeple will be linked to care - earlier in their disease progression, but jumping from PP practice in Kampala to ending TB is too far. Some of the language in the discussion is a bit colloquial as well, and you may want to revisit.

Response: We have reduced the discussion and revised the statements as well as the language.

**Response to Reviewer #2**

Thank you for taking time to read through our work and recognizing the efforts put in to make it better. We have read through the whole document and worked on the formatting.

Kind regards,

Judith Amutuhaire-Ssemasaazi (Corresponding author)
